# Early Cancer Survivorship Distress Trajectories Associated With Socioeconomic Status and Age: Findings From a Multicenter Prospective Study

**DOI:** 10.1002/cam4.71076

**Published:** 2025-08-01

**Authors:** Anja Mehnert‐Theuerkauf, Ute Goerling, Tanja Zimmermann, Jochen Ernst, Myriel Hermann, Beate Hornemann, Ulrich Keilholz, Florian Lordick, Olaf von dem Knesebeck, David Kissane, Anne‐Kathrin Köditz, Franziska Springer

**Affiliations:** ^1^ Department of Medical Psychology and Medical Sociology, Comprehensive Cancer Center Central Germany (CCCG) University Medical Center Leipzig Leipzig Germany; ^2^ Corporate Member of Freie Universität Berlin, Humboldt‐Universität zu Berlin, and Berlin Institute of Health, Charité Comprehensive Cancer Center Charité—Universitätsmedizin Berlin Berlin Germany; ^3^ Department of Psychosomatic Medicine and Psychotherapy Hannover Medical School Hannover Germany; ^4^ National Center for Tumor Diseases Dresden (NCT/UCC) University Hospital Carl Gustav Carus Dresden Dresden Germany; ^5^ National Center for Tumor Diseases (NCT) Berlin Berlin Germany; ^6^ Department of Medicine II (Oncology, Gastroenterology, Hepatology, and Pulmonology), Comprehensive Cancer Center Central Germany (CCCG) University of Leipzig Medical Center Leipzig Germany; ^7^ Institute of Medical Sociology University Medical Center Hamburg‐Eppendorf Hamburg Germany; ^8^ School of Medicine University of Notre Dame Australia Sydney New South Wales Australia; ^9^ Departments of Palliative Care Cabrini Health Melbourne Victoria Australia; ^10^ School of Clinical Sciences Monash Health and Monash University Melbourne Victoria Australia

**Keywords:** age, cancer survivorship, distress, longitudinal study, psycho‐oncology, socioeconomic status

## Abstract

**Background:**

Socioeconomic status' (SES) impact on distress during cancer survivorship has been insufficiently studied, although the consequences of low SES can be cumulative and adversely impact a person's ability to access resources required for improved health and quality of life.

**Patients and Methods:**

We conducted a prospective study involving newly diagnosed patients within 2 months of diagnosis (t1), and at 6‐, 12‐, and 18‐month follow‐up (t2–t4) using the Distress Thermometer (DT). Generalized Linear Mixed Models (GLMM) were used to test for changes in distress over time, with fixed effects of time, SES, and age.

**Results:**

Out of 1702 eligible patients, 965 completed the baseline DT (53% men, 60.5 years); 779, 681, and 626 participated at follow‐ups. Out of 554 completers, 9% were chronically distressed, while 40.8% were never distressed. Distress decreased in 21.3%, increased in 11.0%, and 17.8% fluctuated over time. Low‐SES patients consistently had the highest rates of distress. Distress scores and the frequency of distress (DT ≥ 5) decreased over time in all SES and age groups: For DT mean scores, GLMM revealed a significant effect of time (*χ* ^2^(3) = 72.0, *p* < 0.001), but not of SES (*χ* ^2^(2) = 5.9, *p* = 0.052). For frequency of distress, there was a main effect of time (*χ* ^2^(3) = 41.4, *p* < 0.001) and SES (*χ* ^2^(2) = 15.5, *p* < 0.001). Younger patients (< 65 years) consistently experienced more distress than older patients (≥ 65 years). For DT mean scores, GLMM showed an effect of time (*χ* ^2^(3) = 72.1, *p* < 0.001) and age (*χ* ^2^(1) = 66.2, *p* < 0.001). Similarly, for frequency of distress we found an effect of time (*χ* ^2^(3) = 41.7, *p* < 0.001) and age (*χ* ^2^(1) = 52.8, *p* < 0.001).

**Conclusion:**

Effective psychosocial interventions require a customized approach to decrease distress in vulnerable groups.

**Trial Registration:**

This study was registered in the International Clinical Trials Registry (NCT04620564, https://clinicaltrials.gov/)

## 1 Introduction

Cancer and its treatment are associated with an increased risk of psychological distress. A total of 52% of cancer survivors in different treatment and rehabilitation settings experience high levels of distress [1]. A considerable number of cancer survivors continue to experience distress, and specifically anxiety and depression, for up to 10 years and more after cancer treatment completion [2, 3, 4]. The prevalence of distress in cancer survivors is significantly higher compared to the age‐ and gender‐adjusted general population [2, 4].

The temporal patterns of distress in early survivorship in the first 2 years after diagnosis can be classified into several groups. A substantial proportion of individuals demonstrate resilience, exhibiting stable, low stress levels (73%–80%) [5, 6, 7]. A group of approximately 11%–35% of patients has chronically elevated and high levels of distress [5, 6, 7, 8, 9]. Furthermore, there are groups with a mixed course, including a delayed increase in distress (8%) and a strong change in distress group (8%) [6].

The occurrence and persistence of high distress during cancer treatment and the early survivorship phase has been repeatedly shown to be influenced by a number of risk factors, including younger age [10], female gender [10], being single [11, 12], advanced cancer stages, and poorer performance status [13, 14], as well as pre‐existing psychiatric conditions [13] and ineffective coping strategies [15].

Nevertheless, the potential impact of socioeconomic status (SES) on distress during the cancer trajectory has been largely overlooked, despite evidence indicating that lower income and social deprivation are associated with increased distress [16, 17, 18]. Our own prospective study, on which this publication is based, found no significant influence of SES on mental disorders using the structured diagnostic interview (SCID‐5) for mental disorders based on DSM‐5 criteria shortly after diagnosis. However, we did identify two groups with a higher risk of mental disorders: younger patients with low SES and men with low SES [19]. In addition, patients with low SES consistently showed the highest prevalence rates within the observation period of 18 months, while rates of mental disorders decreased over time in patients with high SES [20].

The early identification of cancer survivors who are at an elevated risk of developing psychological distress is important, particularly in light of the increasing prevalence of cancer in old age groups and the growing population of cancer survivors [21]. This paper builds on our previous paper on the course of mental disorders in the early cancer survivorship period [20], with a particular focus on distress. This is an important area of interest as the number of patients suffering from distress is considerably higher regardless of psychopathology. In addition, the course of distress is of high relevance to the widely established practice of distress screening and psychosocial care [22]. The provision of psycho‐oncological counseling and intervention services tailored to individual needs and resources is of crucial importance for patients experiencing high levels of distress, especially those with fewer educational and socioeconomic resources [23, 24, 25]. Therefore, we aim to analyze the prevalence of psychological distress, stratified by SES and with particular focus on age, and to describe the course of distress over time, from diagnosis to 18 months after early survivorship.

## 2 Material and Methods

### 2.1 Study Design and Participants

We conducted a prospective multi‐center longitudinal observational study involving newly diagnosed cancer patients. Participants underwent assessments at four time points: baseline (t1), 6 months (t2), 12 months (t3), and 18 months (t4). We chose four measurement time points because most previous studies had adopted a cross‐sectional approach, and our objective was to focus on the course of distress at different time points in the early stages of survival. Eligibility criteria included: a diagnosis of a malignant solid tumor (ICD‐10: C00‐C80), age 18 or older, scheduled for treatment at one of the participating study centers, proficient in German, and the physical, mental, and cognitive ability for participation. Exclusion criteria were a second malignancy or cancer relapse. All participants provided written informed consent prior to involvement in the study.

We included three German university comprehensive cancer centers, that is Leipzig, Berlin, and Hannover, as well as cooperating centers in Braunschweig, Dresden, and Göttingen. We obtained ethics approval from the ethics committees at the Medical Faculty of the University of Leipzig, the Technische Universität Dresden, the Hannover Medical School, and the Faculty of Medicine at the University of Göttingen (207/19‐ek, SR‐EK‐536112020, 8533_BO_K_2019, 14/4/21Ü) and registered the study in the International Clinical Trials Registry (NCT04620564). The detailed study protocol has been published [26].

### 2.2 Data Collection

Patients were recruited from April 2020 to July 2022, with data collection ending in January 2024. Eligibility was determined through a systematic review of medical records by the research team. Eligible patients were approached during their inpatient cancer treatment and received written study information. Due to COVID‐19 restrictions, patients received an invitation letter and study materials by post if they could not be contacted directly. Participants were interviewed at all time points (t1–t4) using the SCID‐5 (data published elsewhere [19, 20]), alongside validated questionnaires completed at the cancer center or at home. Reminders for unreturned questionnaires were sent after 2 and 3 weeks.

### 2.3 Measures

Medical data, including type and date of cancer diagnosis, TNM/UICC stage, treatment history, and current cancer treatments, were obtained from medical records and questionnaires. Sociodemographic data was gathered during the SCID‐5 interview. The Winkler Stolzenberg index [27] for SES was calculated using education, income, and occupation data at baseline. SES was categorized into low, medium, and high levels. Physical functionality was evaluated using the Karnofsky performance status [28], ranging from 0 (“dead”) to 100 (“normal, no complaints, and no signs of disease”).

We assessed psychological distress using the validated German version of the NCCN Distress Thermometer (DT) [29]. The DT is a valid, reliable, and widely used screening measure [30]. The screening contains a visual analogue scale with one item ranging from 0 (“no distress”) to 10 (“extreme distress”), which is used to quantify the global level of distress experienced in the past week, including the current day. The standardized 36‐item problem checklist was not used. A score of ≥ 5 on the visual analogue scale is recommended as the cut‐off for a clinically significant level of distress [29].

### 2.4 Statistical Analyses

Baseline sociodemographic and medical characteristics are displayed descriptively. Mean distress values (Distress Thermometer) with standard deviation (SD) were stratified by SES group (low, medium, high), as well as age groups (young < 65 years, old ≥ 65 years). In addition, the frequency of patients with distress values above the clinical cut‐off (DT ≥ 5) was calculated and stratified by SES and age. Generalized Linear Mixed Models (GLMM) were used to test for changes in distress over time, with fixed effects of time, SES, and age, as well as random intercepts for subjects. Time, SES, and age group were treated as a categorical variables. The GLMM were fitted for DT mean values and for the frequency of clinically distressed patients with a binomial distribution, and were run separately for SES and age.

To identify distress trajectory groups, the well‐validated DT cut‐off (≥ 5) was used to determine five different trajectory groups: patients being above the cut‐off at every measurement point (“distressed”), below the cut‐off at every measurement point (“non‐distressed”), patients with decreasing values from baseline (≥ 5) to t4 (< 5) (“decreasing”), increasing values from baseline (< 5) to t4 (≥ 5) (“increasing”), and lastly patients showing fluctuating values above and below the cut‐off across time points (“fluctuating”). Frequency of patients in each trajectory group was calculated with mean DT values over time. Subsequently, multiple logistic regression analyses were performed to identify relevant associated characteristics. Sociodemographic (age, sex, SES, partnership, residential area) and medical characteristics (UICC, treatment, cancer type, physical functionality) were entered in two separate multiple regression models in order to avoid overfitting of the model, and thus controlling for all other factors within the respective group. Thereby, each trajectory group was tested in comparison to all other patients, for example, being in the distressed group compared to all other groups. Model assumptions for multiple logistic regression were assessed by examining multicollinearity using variance inflation factors (VIFs) and checking for influential observations using standardized residuals.

All analyses were performed with R statistics software, version 4.3.1, and all tests were two‐tailed (*α* = 5%).

## 3 Results

### 3.1 Participants

Following an initial eligibility check based on medical records, 3327 patients were identified as eligible. A thorough eligibility check was then carried out by contacting patients either during their inpatient stay or by telephone due to restrictions imposed by the ongoing pandemic. Of these, 1575 patients could not be reached to verify final eligibility for study participation (e.g., language skills). Of the remaining 1702 eligible patients, 1150 (67.6%) were ultimately enrolled in the study.

For a detailed flow‐chart see the previous publication by Goerling et al. [19]. For our analysis, patients without SES value were excluded. A total of 965 patients completed the DT at baseline and were considered for our analyses. Distress data at follow‐ups t2–t4 were provided by 779, 681, and 626 participants, respectively. Complete data at all measurement points is available for 554 participants.

Compared to study participants (*n* = 1150), non‐responders excluded due to insufficient eligibility data (*n* = 1575) were older (60.4 vs. 63.4 years, *p* < 0.001) and more likely to be male (53.9% vs. 58.0%, *p* = 0.04). SES and physical functionality data were not available for this group. Non‐responders who declined participation (*n* = 552) did not differ in sex (*p* = 0.49), but were more likely to have a lower SES (low: 16.6% vs. 27.4%; high: 38.9% vs. 26.8%, *p* < 0.001), were older (60.4 vs. 65.9 years, *p* < 0.001), and had worse physical functionality (80.0 vs. 73.6, *p* < 0.001), compared to study participants [20].

Study completers (*n* = 554) and non‐completers (*n* = 411) did not differ in age (*p* = 0.41), sex (*p* = 0.21), partnership (*p* = 0.54), distress (*p* = 0.23), mental disorder at baseline (*p* = 0.70), and SES (*p* = 0.34), but had a less advanced disease (UICC stage IV: 7.6% vs. 19.8%, *p* < 0.001).

Baseline sample characteristics are presented in Table 1. Sociodemographic differences were found in SES groups, with men having a higher SES (low: 13.4% vs. 15.5%; medium: 41.2% vs. 49.2%; high: 45.3% vs. 35.3%, *p* = 0.006) and participants with a partner having a higher SES (low: 10.3% vs. 29.5%; medium: 43.7% vs. 45.7%; high: 45.9% vs. 24.8%, *p* < 0.001). Although there was a tendency for low SES to be associated with more advanced disease as measured by higher UICC stage, there is no significant difference in UICC stage between the three SES groups (*χ* ^2^(6) = 12.5, *p* = 0.053). Physical functioning (Karnofsky score) was also not predicted by the SES (*F*(1, 771) = 1.5, *p* = 0.22).

**TABLE 1:** Baseline sample characteristics (*n* = 965).

	%	(*n*)
Age, years mean (SD, range)	60.5 (13.1, 21–92)	
Sex		
Men	53.3	(514)
Women	46.7	(451)
Marital status		
Married	66.1	(587)
Single	17.3	(154)
Divorced/separated	10.6	(94)
Widowed	6.0	(53)
Partnership	80.5	(716)
Educational qualification		
University degree	44.1	(395)
High school/vocational training	53.6	(480)
No qualification	2.3	(21)
Occupation		
Employed	50.6	(449)
Retired	44.7	(397)
Unemployed	2.3	(20)
Other	2.5	(22)
Socioeconomic status		
Low	14.4	(139)
Medium	45.0	(434)
High	40.6	(392)
Residential area		
Urban (≥ 20,000 inhabitants)	56.8	(508)
Rural (< 20,000 inhabitants)	43.2	(387)
Tumor site		
Prostate (C61)	19.1	(184)
Skin (C43–C44)	17.3	(167)
Digestive organs (C15–C26)	13.8	(138)
Breast (C50)	13.3	(133)
Female genital organs (C51–C58)	12.8	(128)
Kidney/urinary tract (C64–C68)	7.2	(69)
Head and neck (C00–C14)	5.9	(57)
Lung (C34)	3.2	(31)
Other	7.5	(72)
Months since diagnosis,^a^ mean, median (range)	1.3, 1.0 (0–6)	
≤ 2 months	86.5	(835)
> 2 months	13.5	(130)
UICC stage		
I	43.6	(421)
II	22.0	(212)
III	17.4	(168)
IV	12.2	(118)
Not determinable^b^	4.8	(46)
Cancer treatment^c^		
Surgery	87.3	(819)
Radiotherapy	17.2	(161)
Chemotherapy	20.0	(188)
Other^d^	12.5	(117)
Physical functioning (Karnofsky), mean (SD, range)	80.0 (21.6, 10–100)	

*Note:* (*n*) are valid answers only, with deviations from the full sample size being missing values; percentages are based on valid answers.

Abbreviations: SD, standard deviation; UICC, Union for International Cancer Control disease stage.

^a^ Months since diagnosis in relation to first questionnaire completion. > 2 months: deviation from the study protocol (maximum up to 6 months), since recruitment during COVID‐19 was only possible indirectly via mail, which extended the time required for the study inclusion process.

^b^ Not determinable, for example, basalioma.

^c^ Multiple responses possible; based only on patients who received a cancer treatment (*n* = 938).

^d^ Including hormone or immunotherapy.

### 3.2 Course of Distress Stratified by Socioeconomic Status and Age

**FIGURE 1:**
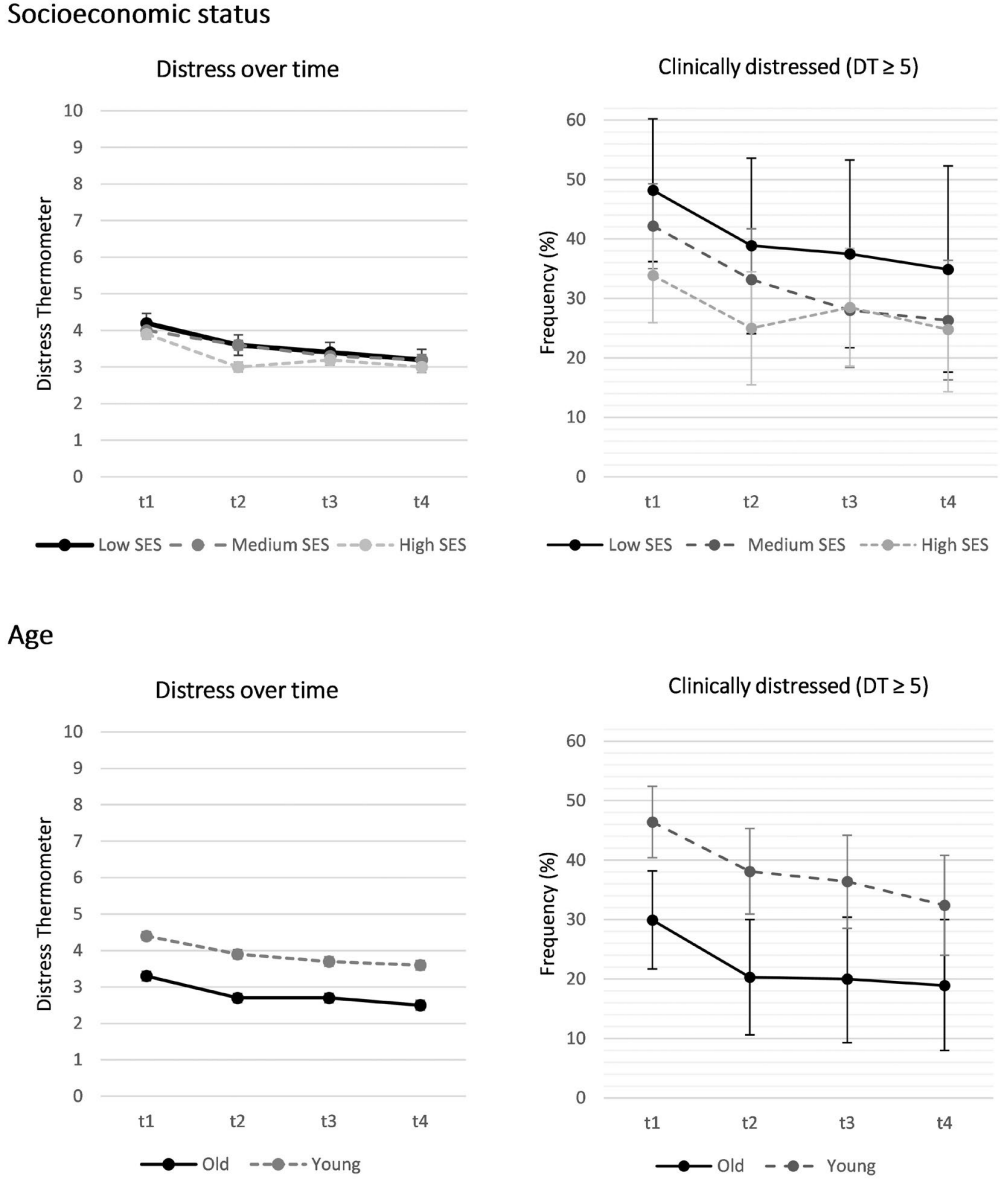
Course of distress stratified by socioeconomic status (SES) and age (mean values with standard error and frequencies with 95% confidence interval).

Distress values and the frequency of distressed patients (≥ 5) decrease over time in all SES and age groups. The group with low SES consistently shows the highest rates of distress (Figure 1, Table S1). For the DT mean values, the GLMM revealed a significant effect of time (*χ* ^2^(3) = 72.0, *p* < 0.001), but not SES (*χ* ^2^(2) = 5.9, *p* = 0.052). For the frequency of clinically distressed patients, there was a main effect of time (*χ* ^2^(3) = 41.4, *p* < 0.001) and SES (*χ* ^2^(2) = 15.5, *p* < 0.001).

Younger patients (< 65 years) consistently show higher levels of distress than older patients (≥ 65 years). For the DT mean values, the GLMM indicated an effect of time (*χ* ^2^(3) = 72.1, *p* < 0.001) and age (*χ* ^2^(1) = 66.2, *p* < 0.001), as well as for frequency of clinically distressed patients (time: *χ* ^2^(3) = 41.7, *p* < 0.001; age: *χ* ^2^(1) = 52.8, *p* < 0.001). For detailed coefficients in comparison to reference groups, see Table S2. Sensitivity analyses with regard to psychological distress over time, stratified by partnership and gender, are shown in Table S3.

### 3.3 Distress Trajectories

**FIGURE 2:**
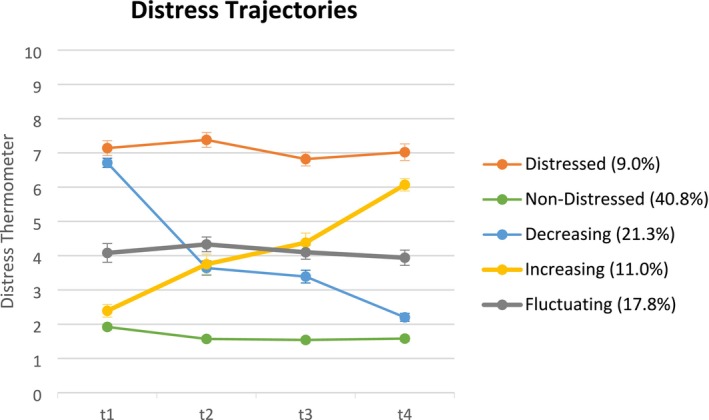
Distress trajectory groups (mean values and standard error).

The following analyses were carried out among study completers with DT data at every measurement point (*n* = 554). In total, 50 patients (9.0%) are clinically *distressed* (≥ 5) at every measurement point, and 226 patients (40.8%) are *non‐distressed* (< 5) at every measurement point. *Decreasing* values from baseline (≥ 5) to t4 (< 5) are observed in 118 patients (21.3%), and *increasing* values from baseline to t4 in 61 patients (11.0%). The remaining 99 patients (17.8%) *fluctuate* between values above and below the cut‐off across time points. Figure 2 displays mean DT values of the five trajectory groups.

Multiple logistic regression analyses were performed for individual trajectory groups regarding relevant sociodemographic and medical factors (Table 2). Patients in the continuously *distressed* group were more likely to be younger, have advanced cancer stage, and have worse physical functionality. Patients in the continuously *non‐distressed* group were older, had higher SES, and were more likely to have prostate cancer. Patients in the *decreasing* distress group were more likely to be younger, did not undergo radiotherapy, and were less likely to have skin cancer. *Increasing* distress was more likely to occur in patients with kidney cancer. Patients with *fluctuating* distress values were more likely to have no partner and receive other cancer therapy than surgery, radiation, and chemotherapy.

**TABLE 2:** Multiple logistic regression analysis on distress trajectory groups.

	Distressed *n* = 50			Non‐distressed *n* = 226			Decreasing *n* = 118			Increasing *n* = 61			Fluctuating *n* = 99		
	b (SE)	*p*	OR (95% CI)	b (SE)	*p*	OR (95% CI)	b (SE)	*p*	OR (95% CI)	b (SE)	*p*	OR (95% CI)	b (SE)	*p*	OR (95% CI)
Sociodemographic															
Age	−0.05 (0.01)	**< 0.001**	0.96 (0.93–0.98)	0.05 (0.01)	**< 0.001**	1.05 (1.04–1.07)	−0.03 (0.01)	**< 0.001**	0.97 (0.96–0.99)	0.00 (0.01)	0.99	1.00 (0.98–1.02)	−0.01 (0.01)	0.15	0.99 (0.97–1.00)
Sex^a^	0.35 (0.33)	0.29	1.42 (0.75–2.76)	−0.25 (0.20)	0.20	0.78 (0.53–1.15)	−0.17 (0.23)	0.45	0.84 (0.54–1.31)	0.44 (0.30)	0.15	1.55 (0.86–2.85)	0.15 (0.24)	0.52	1.17 (0.73–1.88)
SES	−0.25 (0.23)	0.28	0.78 (0.50–1.23)	0.40 (0.15)	**0.006**	1.49 (1.12–1.98)	−0.15 (0.16)	0.35	0.86 (0.63–1.18)	0.03 (0.22)	0.88	1.03 (0.68–1.60)	−0.32 (0.17)	0.06	0.73 (0.52–1.02)
Partnership^b^	0.24 (0.36)	0.50	1.27 (0.61–2.55)	−0.01 (0.25)	0.98	0.99 (0.61–1.61)	0.43 (0.26)	0.09	1.54 (0.92–2.55)	−0.20 (0.38)	0.60	0.82 (0.37–1.66)	−0.72 (0.33)	**0.03**	0.49 (0.24–0.91)
Residential area^c^	0.01 (0.32)	0.98	1.01 (0.54–1.92)	0.31 (0.19)	0.11	1.36 (0.93–1.98)	−0.18 (0.22)	0.42	0.84 (0.54–1.29)	−0.10 (0.29)	0.74	0.91 (0.51–1.62)	−0.22 (0.23)	0.33	0.80 (0.51–1.26)
Medical															
UICC	0.52 (0.19)	**0.01**	1.68 (1.16–2.47)	−0.20 (0.14)	0.14	0.81 (0.63–1.06)	−0.22 (0.15)	0.14	0.08 (0.59–1.07)	−0.10 (0.20)	0.62	0.91 (0.61–1.32)	0.25 (0.16)	0.13	1.28 (0.93–1.77)
Treatment															
Surgery	−0.76 (0.56)	0.17	0.47 (0.16–1.47)	0.71 (0.41)	0.08	2.04 (0.94–4.69)	−0.65 (0.41)	0.11	0.52 (0.23–1.19)	0.00 (0.59)	0.99	1.00 (0.33–1.32)	0.42 (0.50)	0.41	1.52 (0.59–4.31)
Chemotherapy	−1.01 (0.52)	0.051	0.36 (0.12–0.95)	0.50 (0.33)	0.13	1.65 (0.87–3.13)	0.43 (0.34)	0.21	1.54 (0.78–3.00)	−0.25 (0.48)	0.61	0.78 (0.29–1.93)	−0.42 (0.40)	0.29	0.66 (0.29–1.40)
Radiotherapy	0.37 (0.53)	0.49	1.45 (0.49–3.99)	0.51 (0.34)	0.13	1.66 (0.86–3.24)	−1.00 (0.44)	**0.02**	0.37 (0.15–0.83)	−0.82 (0.62)	0.19	0.44 (0.11–1.35)	0.48 (0.40)	0.23	1.62 (0.73–3.51)
Other therapy	0.22 (0.55)	0.68	1.25 (0.40–3.50)	−0.15 (0.38)	0.69	1.86 (0.39–1.80)	−0.33 (0.45)	0.46	0.72 (0.28–1.67)	−1.32 (0.77)	0.09	0.27 (0.04–0.99)	0.86 (0.41)	**0.03**	2.37 (1.05–5.21)
Cancer type^d^															
Prostate	−1.20 (0.83)	0.15	0.30 (0.05–1.57)	1.36 (0.49)	**0.006**	3.9 (1.51–10.66)	−0.94 (0.55)	0.09	0.39 (0.13–1.18)	0.70 (1.13)	0.53	2.02 (0.30–40.02)	−0.98 (0.70)	0.16	0.38 (0.09–1.51)
Skin	0.45 (0.75)	0.55	1.57 (0.38–7.61)	0.49 (0.50)	0.32	1.64 (0.63–4.47)	−1.30 (0.59)	**0.03**	0.27 (0.09–0.87)	1.19 (1.09)	0.28	3.28 (0.56–62.64)	−0.10 (0.64)	0.88	0.91 (0.27–3.39)
Digestive organs	−0.56 (0.77)	0.47	0.57 (0.12–2.75)	0.07 (0.53)	0.89	1.08 (0.38–3.11)	−0.53 (0.57)	0.35	0.59 (0.19–1.84)	1.65 (1.12)	0.14	5.19 (0.80–102.57)	0.26 (0.64)	0.68	1.30 (0.38–4.84)
Breast	−0.55 (0.78)	0.48	0.58 (0.13–2.85)	−0.04 (0.51)	0.94	0.96 (0.36–2.70)	−0.55 (0.55)	0.31	0.58 (0.20–1.73)	1.87 (1.10)	0.0	6.51 (1.09–125.24)	0.10 (0.63)	0.88	1.10 (0.33–4.06)
Female genital organs	−0.12 (0.72)	0.86	0.88 (0.22–4.01)	−0.49 (0.52)	0.35	0.61 (0.22–1.75)	−0.21 (0.54)	0.70	0.81 (0.29–2.40)	1.65 (1.10)	0.13	5.22 (0.8–101.29)	0.29 (0.62)	0.64	1.34 (0.41–4.86)
Kidney/urinary tract	0.15 (0.90)	0.87	1.16 (0.18–6.77)	−0.51 (0.71)	0.47	0.60 (0.14–2.34)	−0.25 (0.70)	0.72	0.78 (0.19–3.02)	2.54 (1.17)	**0.03**	12.71 (1.72–263.38)	−1.14 (1.18)	0.22	0.24 (0.01–1.79)
Lung	−1.17 (1.31)	0.37	0.31 (0.01–3.29)	0.39 (0.92)	0.67	1.47 (0.22–8.91)	−0.66 (1.20)	0.58	0.51 (0.02–4.19)	2.15 (1.56)	0.17	8.60 (0.28–269.43)	0.02 (1.03)	0.98	1.02 (0.11–7.26)
Other	−0.42 (0.85)	0.62	0.66 (0.12–3.56)	−0.30 (0.63)	0.63	0.74 (0.21–2.52)	0.28 (0.62)	0.65	1.32 (0.39–4.58)	1.59 (1.21)	0.19	4.89 (0.55–105.54)	−0.67 (0.81)	0.41	0.51 (0.09–2.46)
Physical functionality	−0.02 (0.01)	**0.01**	0.98 (0.96–0.99)	0.01 (0.01)	0.28	1.01 (0.99–1.02)	−0.01 (0.01)	0.43	0.99 (0.98–1.02)	0.01 (0.01)	0.24	1.01 (0.99–1.03)	0.01 (0.01)	0.44	1.01 (0.99–1.02)

*Note:* Multiple logistic regression analysis, with separate models for sociodemographic and medical characteristics; analysis performed for each group separately compared to all others (e.g., distressed (*n* = 50) versus all other groups (*n* = 504)); significant values marked in bold.

Abbreviations: 95% CI, 95% confidence interval; OR, odds ratio; SE, standard error.

^a^ Reference group men.

^b^ Reference group partner.

^c^ Reference group urban.

^d^ Reference group head and neck cancer.

## 4 Discussion

Our results suggest that, independent of SES and age, initial distress is reduced in the early survivorship phase of the first year and a half after cancer diagnosis in the overall sample of primary cancer survivors. In the initial 2 months following diagnosis, 40% of patients exhibited elevated levels of distress, compared to 27% after 18 months. Distress levels are highest around diagnosis and tend to decrease over time, which may reflect the patient's natural psychological adjustment to the cancer diagnosis as well as adequate social support. Our findings could be further explained by the establishment of evidence‐based psycho‐oncological care in the Comprehensive Cancer Centers of Germany over recent years as part of the National Cancer Plan. Consequently, low‐threshold psychosocial care is available for all cancer survivors. In accordance with the national care guidelines for psycho‐oncology [31], every patient should be screened as part of their cancer treatment and receive needs‐based inpatient psychosocial care [31]. Previous studies have demonstrated the efficacy of these screening procedures [32, 33]. Outpatients also have access to complimentary psychosocial cancer counseling [34, 35] and oncological rehabilitation, encompassing psycho‐oncological support. However, it is crucial to recognize the potential influence of sampling bias, as participation and completion of the study may have been disproportionately affected by a higher proportion of patients with high SES. This suggests that the patients who participated and remained in the study possessed greater educational and socioeconomic resources, which could account for the significant decrease in distress observed.

Despite the decline in overall distress levels within our sample, survivors with low SES and those who are younger had the highest rates of distress observed among participants during the early stages of survival. In terms of socioeconomic status, there is a substantial body of evidence indicating that low SES is associated with adverse health outcomes [36, 37, 38, 39]. Populations with low income experience higher rates of mental illness, chronic health conditions, and substance use disorders [40, 41, 42]. The mechanisms by which socioeconomic status affects health outcomes are multifaceted, involving behavioral, environmental, and biological factors. In our study, some of these factors could also explain why the level of distress in patients with low SES is higher than in patients with medium and high SES at all measurement times. These factors include, but are not limited to, a lack of health literacy as well as ineffective health behavior (e.g., smoking, excessive alcohol consumption, poor diet, and obesity) [43, 44]. Additionally, the lack of physical activity, elevated stress levels, and a higher prevalence of adverse life events have been identified as contributing factors [45, 46, 47]. Researchers also suggest that the consequences of disadvantaged SES may be cumulative and have a prospective effect on an individual's capacity to access social and economic resources [48, 49], such as the utilization of psychosocial support services.

Considering the increased psychological distress observed in younger patients throughout the course of the disease in the early stages of cancer survival, our findings support the observations of previous studies [50, 51]. It is widely documented that younger patients often have higher levels of psychological vulnerability compared to their older counterparts. This increased vulnerability is the result of a combination of biological, psychological, and environmental factors, such as increased susceptibility to stress and mental disorders, increased sensitivity to stressors, and difficulties in emotion regulation and coping.

The present study also reveals an interesting result in relation to clinically distressed patients with high SES, who show a slight increase in distress 1 year after cancer treatment. Possible explanations for this could be the patients' higher expectations of their functionality in everyday life and at work. It can be assumed that these expectations potentially increase the perception of existing problems due to fears of decline and loss with regard to autonomy, prosperity, mobility, and independence.

Our study demonstrated that 9% of patients exhibited clinical distress at each measurement point during the early survivorship period. Patients within this distress group were more likely to be younger, to have a more advanced stage of cancer, and to demonstrate poorer physical functioning. The percentage observed in our study is marginally lower than the numbers reported in comparable studies, which indicate that 11%–35% of patients exhibit chronically elevated and high levels of distress [5, 6, 7, 8, 9]. The aforementioned sample bias with regard to higher SES could contribute to the lower number of chronically distressed patients. Another possible explanatory factor could be the relatively high number of patients at an early tumor stage and the high number of prostate cancer patients in our sample, who often have significantly lower distress than other patient groups [52]. However, it is crucial to acknowledge that an additional 11% of patients experience an increase in distress, indicating that 20% of patients can be categorized as particularly vulnerable and in need of professional psychosocial support.

It is noteworthy, however, that up to 41% of patients, and in particular prostate cancer patients, never show a high level of distress in the early survival phase. This finding also emphasizes the influence of advanced age and high SES. This group appears to have sufficient resources and adaptive coping styles, which appear to contribute to a persistently low level of distress.

The observation that men had higher SES compared to women, and that those with partners had a higher SES, reveals critical social dynamics. Differences in SES are likely to have a significant effect with gender on health outcomes in cancer populations. These disparities manifest in differential screening rates, access to healthcare, variations in health behaviors, and differences in disease progression, which collectively contribute to unequal outcomes in cancer incidence rates and cancer mortality [37, 38, 53]. This intersectionality highlights the additional disadvantages faced by socioeconomically disadvantaged women, but also men, who have a higher cancer mortality rate compared to people with higher SES. However, the interactions between gender, partnership, SES, and cancer outcome still need to be better understood, particularly with regard to the survivorship phase. It is also important that social inequalities in cancer aftercare need to be addressed in all aspects of cancer and follow‐up care, with measures to reduce incidence and prolong survival [54]. Such measures include systematic screening and symptom management in cancer aftercare [37], but also better public health and prevention approaches to improve lifestyles in vulnerable and disadvantaged groups.

Addressing social inequalities in psycho‐oncology care requires a multi‐faceted approach that addresses inequalities in access, awareness, and adherence to treatment. Proposed solutions include developing and implementing formal guidelines and policies to standardize psycho‐oncology support across healthcare systems, conducting routine screening for mental health problems, addressing the stigma of mental illness, and increasing the inclusion of the psychosocial dimensions of cancer care in oncology education and interprofessional care models [55]. Targeted interventions should focus on providing additional support to lower socioeconomic groups to improve adherence and outcomes [56].

Meta‐analyses show significant small‐to‐medium effects of individual and group psychotherapy and psychoeducation on emotional distress and quality of life in adult cancer patients, with these effects partially sustained in the medium term across various cancer populations [57]. There is also evidence of small but beneficial effects on psychosocial outcomes for vulnerable groups such as immigrant and limited language proficient cancer patients [58].

A major strength of our study is its longitudinal design, which captures early survival within 2 months of cancer diagnosis and follows patients up to 1.5 years after initial cancer diagnosis, with a comparatively high retention rate. Our study had recruitment difficulties due to COVID‐19 restrictions, which were particularly evident in people with low SES.

Given the results of our non‐responder and drop‐out analyses and the deviations from cancer prevalence rates for certain cancer types (e.g., lung, breast and skin cancer), our results should be interpreted with caution, and further studies are needed to validate our findings. The non‐responder analyses included patients who were excluded as non‐responders due to insufficient eligibility data and patients who denied their participation. In terms of socio‐demographic characteristics, non‐responders had a higher age, a higher male prevalence, lower SES, and limited physical functionality, as well as a more advanced disease in non‐completers. These factors can lead to various, partly opposing biases. It can be deduced that the sample is biased with regard to younger women with better physical functionality. Since distress is higher in younger patients and women than in older patients and men, the sample could overestimate distress on the one hand [1, 59, 60]. In contrast, distress tends to be lower with improved physical functionality and less advanced disease [61], which could potentially contribute to an underestimation of distress in our sample. As a consequence, the bias effect with regard to socio‐demographics and physical functioning in our study is not clear.

In future studies, greater efforts should be made to include vulnerable groups, such as patients of low social status, in a more representative way. It is incumbent on researchers to develop strategies to mitigate missing data, such as the use of mixed methods or advanced statistical techniques to estimate the impact of non‐responders.

## 5 Conclusions

Our findings provide significant indications for the design of psychosocial support services, both face‐to‐face and web‐based. The design of psychosocial support services for cancer patients with low education and low SES requires a customized approach that addresses their specific needs and barriers. Effective interventions should focus on accessibility, education, and community involvement to improve the quality of life of these patients. Services should be easily accessible and minimize barriers such as transport and cost. Community‐based programs can facilitate this access. Implementing person‐centered care ensures that services are responsive to individual needs, preferences, and circumstances, which is essential for patients with low social status [62]. Providing clear, understandable information about cancer and support options is crucial. Educational material should be tailored to the patients' level of knowledge [58, 63]. Programs that educate patients about coping strategies and available resources can encourage them to seek help [63]. Involving family members and community resources can strengthen support networks, which is particularly beneficial for patients from socially disadvantaged backgrounds. Services should be culturally relevant and responsive to the unique challenges of specific populations to ensure inclusion and respect. While these strategies can significantly improve psychosocial support for low‐income cancer patients, it is also important to recognize that systemic barriers, such as healthcare inequalities and stigma [64], may continue to hinder access to these services.

## Author Contributions

**Anja Mehnert‐Theuerkauf:** conceptualization (lead), data curation (supporting), formal analysis (supporting), funding acquisition (lead), investigation (lead), methodology (lead), project administration (lead), resources (lead), software (supporting), validation (lead), visualization (equal), writing – original draft (lead), writing – review and editing (equal). **Ute Goerling:** conceptualization (equal), funding acquisition (equal), writing – review and editing (equal). **Tanja Zimmermann:** conceptualization (equal), funding acquisition (equal), writing – review and editing (equal). **Jochen Ernst:** conceptualization (equal), data curation (equal), funding acquisition (equal), writing – review and editing (equal). **Myriel Hermann:** writing – review and editing (equal). **Beate Hornemann:** writing – review and editing (equal). **Ulrich Keilholz:** resources (equal), writing – review and editing (equal). **Florian Lordick:** resources (equal), writing – review and editing (equal). **Olaf von dem Knesebeck:** supervision (equal), writing – review and editing (equal). **David Kissane:** supervision (equal), writing – review and editing (equal). **Anne‐Kathrin Köditz:** writing – review and editing (equal). **Franziska Springer:** data curation (equal), formal analysis (lead), writing – original draft (lead), writing – review and editing (lead).

## Conflicts of Interest

The authors declare no conflicts of interest.

## Supporting information


**Data S1:** cam471076‐sup‐0001‐DataS1.docx.

## Data Availability

The data that support the findings of this study are available from the corresponding author upon reasonable request.
