# Supplementary material for: Early Cancer Survivorship Distress Trajectories Associated With Socioeconomic Status and Age: Findings From a Multicenter Prospective Study
Source: Cancer Med. 2025 Aug 1;14(15):e71076. doi: 10.1002/cam4.71076 (PMC12314646; doi:10.1002/cam4.71076)
Supplement: Supplementary file 1 — Data S1: cam471076‐sup‐0001‐DataS1.docx. [file CAM4-14-e71076-s001.docx]

**Supporting information**

| **SuppInfo table S1**. Psychological distress over time, stratified by socioeconomic status (SES) and age | | | | | | | | |
| --- | --- | --- | --- | --- | --- | --- | --- | --- |
|  | t1  (Baseline) | | t2  (6 months) | | t3  (12 months) | | t4  (18 months) | |
| **Distress (Distress Thermometer, DT)** | | | | | | | | |
|  | n | M (SD) | n | M (SD) | n | M (SD) | n | M (SD) |
| All participants | 965 | 3.9 (2.7) | 779 | 3.4 (2.5) | 681 | 3.3 (2.4) | 626 | 3.1 (2.5) |
| **Socioeconomic status (SES)** | | | | | | | | |
| Low SES | 139 | 4.2 (3.1) | 108 | 3.6 (2.9) | 96 | 3.4 (2.7) | 83 | 3.2 (2.6) |
| Medium SES | 434 | 4.0 (2.7) | 355 | 3.6 (2.5) | 304 | 3.3 (2.3) | 281 | 3.2 (2.4) |
| High SES | 392 | 3.8 (2.6) | 316 | 3.0 (2.4) | 281 | 3.2 (2.5) | 262 | 3.0 (2.5) |
| **Age** | | | | | | | | |
| Old (≥ 65) | 394 | 3.3 (2.6) | 325 | 2.7 (2.2) | 285 | 2.7 (2.2) | 259 | 2.5 (2.2) |
| Young (< 65) | 571 | 4.4 (2.8) | 454 | 3.9 (2.6) | 396 | 3.7 (2.5) | 367 | 3.6 (2.5) |
| **Clinically distressed (DT ≥ 5)** | | | | | | | | |
|  | n | % [CI] | n | % [CI] | n | % [CI] | n | % [CI] |
| All participants | 383 | 39.7 | 239 | 30.7 | 201 | 29.5 | 168 | 26.8 |
| **Socioeconomic status (SES)** | | | | | | | | |
| Low SES | 67 | 48.2  [36.2-60.2] | 42 | 38.9 [24.1-53.6] | 36 | 37.5 [21.7-53.3] | 29 | 34.9 [17.6-52.3] |
| Medium SES | 183 | 42.2 [35.0-49.3] | 118 | 33.2 [24.7-41.7] | 85 | 28.0 [18.4-37.5] | 74 | 26.3 [16.3-36.4] |
| High SES | 133 | 33.9 [25.9-42.0] | 79 | 25.0 [15.5-34.5] | 80 | 28.5 [18.6-38.4] | 65 | 24.8 [14.3-35.3] |
| **Age** | | | | | | | | |
| Old (≥ 65) | 118 | 29.9 [21.7-38.2] | 66 | 20.3 [10.6-30.0] | 57 | 20.0 [9.6-30.4] | 49 | 18.9 [8.0-30.0] |
| Young (< 65) | 265 | 46.4 [40.4-52.4] | 173 | 38.1 [30.9-45.3] | 144 | 36.4 [28.5-44.2] | 119 | 32.4 [24.0-40.8] |
|  | | | | | | | | |

| **SuppInfo table S2.** Regression coefficients and standard errors (SE) from Generalized Linear Mixed Models (GLMM) for fixed effects of time, socioeconomic status (SES) and age | | | |
| --- | --- | --- | --- |
|  | **Contrast** | ***β*** | **SE** |
| **Distress (Distress Thermometer, DT)** | | | |
| Time | t2 vs. t1 | -0.56 | 0.09 |
|  | t3 vs. t1 | -0.60 | 0.10 |
|  | t4 vs. t1 | -0.74 | 0.10 |
| SES | middle vs. low | -0.12 | 0.20 |
|  | high vs. low | -0.41 | 0.21 |
| Age | young vs. old | 1.07 | 0.13 |
| **Clinically distressed (DT ≥ 5)** | | | |
| Time | t2 vs. t1 | -0.56 | 0.13 |
|  | t3 vs. t1 | -0.62 | 0.13 |
|  | t4 vs. t1 | -0.78 | 0.14 |
| SES | middle vs. low | -0.46 | 0.22 |
|  | high vs. low | -0.84 | 0.22 |
| Age | young vs. old | 1.08 | 0.15 |
| Note. Coefficients are displayed in comparison to respective reference group (time: t1, SES: low, age: old).  Abbreviations: Socioeconomic status (SES), Distress Thermometer (DT), standard error (SE) | | | |

| **SuppInfo table S3**. Subgroup analysis - psychological distress over time, stratified by partnership and gender | | | | | | | | |
| --- | --- | --- | --- | --- | --- | --- | --- | --- |
|  | **t1**  **(Baseline)** | | **t2**  **(6 months)** | | **t3**  **(12 months)** | | **t4**  **(18 months)** | |
| **Partner** | | | | | | | | |
| **Distress Thermometer** | | | | | | | | |
|  | n | M (SD) | n | M (SD) | n | M (SD) | n | M (SD) |
| Partner | 716 | 3.9 (2.7) | 594 | 3.3 (2.5) | 515 | 3.3 (2.4) | 486 | 3.1 (2.5) |
| No partner | 173 | 4.5 (3.0) | 151 | 3.6 (2.7) | 138 | 3.3 (2.3) | 116 | 3.2 (2.4) |
| Men | 514 | 3.4 (2.5) | 412 | 3.0 (2.3) | 348 | 2.9 (2.2) | 334 | 2.7 (2.2) |
| Women | 451 | 4.6 (2.9) | 367 | 3.8 (2.6) | 333 | 3.7 (2.5) | 292 | 3.6 (2.6) |
| **Clinically distressed (DT ≥ 5)** | | | | | | | | |
|  | n | % [95% CI] | n | % | n | % | n | % |
| Partner | 267 | 37.7 [31.5-43.1] | 173 | 29.1 [22.4-35.9] | 152 | 29.5 [22.3-36.8] | 126 | 25.9 [18.3-33.6] |
| No partner | 89 | 51.4 [41.1-61.8] | 58 | 38.4 [25.9-50.9] | 40 | 29.0 [14.9-43.0] | 33 | 28.4 [13.1-43.8] |
| Men | 169 | 32.9 [25.8-40.0] | 102 | 24.8 [16.4-33.1] | 83 | 23.9 [14.7-33.0] | 70 | 21.0 [11.4-30.5] |
| Women | 214 | 47.5 [40.8-54.1] | 137 | 37.3 [29.2-45.4] | 118 | 35.4 [26.8-44.1] | 98 | 33.6 [24.2-42.9] |
|  | | | | | | | | |
